# Supplementary material for: Prevalence and Factors of Osteoporosis and High Risk of Osteoporotic Fracture in Patients with Ankylosing Spondylitis: A Multicenter Comparative Study of Bone Mineral Density and the Fracture Risk Assessment Tool
Source: J Clin Med. 2022 May 17;11(10):2830. doi: 10.3390/jcm11102830 (PMC9146147; doi:10.3390/jcm11102830)
Supplement: Supplementary file 1 [file jcm-11-02830-s001.zip › jcm-1697573-supplementary.pdf]

Supplementary Table S1 Univariate analysis of variables associated with high-risk of fracture based on the FRAX with and without BMD and osteoporosis based on the WHO criteria

	FRAX with BMD		FRAX without BMD		Osteoporosis of the WHO	
	OR (95% CI)	p-value	OR (95% CI)	p-value	OR (95% CI)	p-value
Age	1.08 (1.03, 1.14)	**0.002**	1.2 (1.1, 1.3)	**< 0.001**	1.02 (0.99, 1.04)	0.24
Sex (Men)	0.86 (0.34, 2.23)	0.462	0.2 (0.08, 0.55)	**0.002**	0.7 (0.35, 1.34)	0.299
Menopause	4.6 (1.46, 14)	**0.003**	5.4 (2, 16.2)	**< 0.001**	4.5 (2.54, 8.36)	**< 0.001**
BMI < 25kg/m^2	0.97 (0.92, 1.12)	0.205	0.93 (0.89, 0.98)	**0.004**	1.79 (0.69, 3.12)	0.108
Disease duration	1 (0.88, 1.16)	0.921	0.91 (0.79, 1)	0.165	0.942 (0.86, 1.04)	0.215
Smoking	1.45 (0.48, 4.4)	0.512	2.71 (0.59, 12.3)	0.198	1.05 (0.48, 2.32)	0.905
Alcohol use	1.01 (0.34, 2.89)	0.997	1.15 (0.39, 3.34)	0.805	1.45 (0.643, 3.47)	0.358
HLA-B27 positivity	3.96 (0.5, 31.3)	0.192	4.18 (0.8, 77.1)	0.174	8.29 (1.1, 62.8)	**0.041**
ESR	1.03 (0.98, 1.06)	**0.066**	3.2 (0.89, 11.5)	**0.075**	1.67 (1.08, 4.02)	**0.027**
CRP	2.31 (0.72, 7.4)	0.158	3.78 (1.19, 16.8)	**0.041**	2.3 (1.2, 4.62)	**0.015**
Syndesmophyte	0.69 (0.26, 1.8)	0.455	1.56 (0.62, 3.59)	**0.094**	0.93 (0.57, 2.22)	0.729
mSASSS	1.02 (0.99, 1.04)	0.155	1.02 (0.99, 1.04)	**0.067**	0.99 (0.97, 1.01)	0.291
Glucocorticoid use	2.61 (1, 6.79)	**0.05**	2.62 (1.06, 6.57)	**0.036**	1.26 (0.42, 3.15)	**0.055**
NSAIDs use	0.382 (0.05, 3.07)	0.366	0.735 (0.16, 3.47)	0.697	0.346 (0.08, 1.54)	0.163
Biologics use	4.39 (1.5, 12.8)	**0.007**	1.34 (0.55, 3.32)	0.515	2.27 (1.15, 4.47)	**0.018**
PPI use	1.3 (0.43, 3.81)	0.651	2.66 (1.06, 6.67)	**0.035**	1.59 (0.6, 3.8)	0.299
Vitamin D use	0.43 (0.13, 1.41)	0.164	0.82 (0.32, 2.12)	0.687	0.32 (0.13, 0.79)	**0.013**
Calcium use	0.57 (0.19, 1.71)	0.315	0.53 (1.09, 1.43)	0.208	0.403 (0.18, 0.89)	**0.024**

FRAX, Fracture Risk Assessment Tool (%, 10-year probability of major osteoporotic and vertebral fracture, respectively, corresponding to patients aged ≥40 years); BMD, Bone mineral density; WHO, World Health Organization, OR, odds ratio; CI, confidence interval; BMI, body mass index; HLA, human leukocyte antigen; ESR, erythrocyte sedimentation rate; CRP, C-reactive protein; mSASSS, modified Stoke Ankylosing Spondylitis Score; NSAIDs, non-steroidal anti-inflammatory drugs; PPI, proton pump inhibitor. Bold statistics denote p -value ≤ 0.1.

Supplementary Table S2 Comparison of previous fractures-adjusted FRAX between patients with ankylosing spondylitis with fracture and those without

	Fracture (N = 16)	Non-fracture (N = 136)	p-value
High-risk in the FRAX with BMD, N (%)	1 (6.3)	16 (11.8)	0.132
High-risk in the FRAX without BMD, N (%)	4 (25)	12 (2.9)	0.042*

FRAX, fracture risk assessment tool (%, 10-year probability of major osteoporotic, and vertebral fracture respectively, corresponding to patients aged 40 years or older); BMD, bone mineral density. *P < 0.05.
